# What can be learned from high-resolution sleep data using ECoG

**DOI:** 10.1186/1471-2202-13-S1-P94

**Published:** 2012-07-16

**Authors:** Vera M Dadok, Andrew J Szeri, Heidi Kirsch, Jamie Sleigh, Rochelle Zak, Beth Lopour

**Affiliations:** 1 Department of Mechanical Engineering, University of California, Berkeley, CA 94720, USA; 2 Center for Neural Engineering and Prostheses, UC Berkeley and UC San Francisco, CA, USA; 3 School of Medicine, University of California, San Francisco, CA, 94143 USA; 4 School of Medicine, University of Auckland, Grafton, Auckland, 1142, New Zealand; 5 Sleep Disorders Center at UCSF, San Francisco, CA, 94143, USA; 6 Department of Neurobiology, University of California, Los Angeles, CA, 90095, USA

## 

Electrocorticography (ECoG) signals measured from electrodes under the scalp, skull, and dura have higher resolution than scalp electroencephalograph (EEG) recordings due to the reduced thickness of heterogeneous tissue between the cortex and the electrode. Owing to the non-invasive nature of EEG measurements, most studies of the electrical signals generated during sleep make use of EEG. Classically these data, and other concurrent signals (e.g. electromyogram, electrooculogram), are used to classify the sleeping brain state into sleep stages. These stages were originally developed by scientists noticing patterns in early EEG studies. This approach is powerful. However, owing to the electrode placement on the scalp, signal information is lost between the cortex and electrode.

This raises the obvious question, how would sleep staging have evolved differently if ECoG data had been used rather than EEG? Put differently, what are the characteristics or attributes of ECoG signals associated with different sleep stages determined from EEG? This work will compare EEG-based sleep stages to sleep structure seen in simultaneously collected ECoG data, thereby clarifying the extent to which EEG captures the complexity of cortical activity during sleep. Comparisons will be discussed for many measures, including temporal local phenomena (e.g. spindles, accounting for regional influences) and frequency content. This work will also offer suggestions for the interpretation of ECoG data in a way relevant to and extending beyond conventional sleep cycles.

